# Effect of optical blurring of X-ray source on breast tomosynthesis image quality: Modulation transfer function, anatomical noise power spectrum, and signal detectability perspectives

**DOI:** 10.1371/journal.pone.0267850

**Published:** 2022-05-19

**Authors:** Changwoo Lee, Jongduk Baek

**Affiliations:** 1 Medical Metrology Team, Safety Measurement Institute, Korea Research Institute of Standards and Science (KRISS), Daejeon, South Korea; 2 School of Integrated Technology, Yonsei University, Incheon, South Korea; US Department of Agriculture, UNITED STATES

## Abstract

We investigated the effect of the optical blurring of X-ray source on digital breast tomosynthesis (DBT) image quality using well-designed DBT simulator and table-top experimental systems. To measure the in-plane modulation transfer function (MTF), we used simulated sphere phantom and Teflon sphere phantom and generated their projection data using two acquisition modes (i.e., step-and-shoot mode and continuous mode). After reconstruction, we measured the in-plane MTF using reconstructed sphere phantom images. In addition, we measured the anatomical noise power spectrum (aNPS) and signal detectability. We constructed simulated breast phantoms with a 50% volume glandular fraction (VGF) of breast anatomy using the power law spectrum and inserted spherical objects with 1 mm, 2 mm, and 5 mm diameters as breast masses. Projection data were acquired using two acquisition modes, and in-plane breast images were reconstructed using the Feldkamp-Davis-Kress (FDK) algorithm. For the experimental study, we used BR3D breast phantom with 50% VGF and obtained projection data using a table-top experimental system. To compare the detection performance of the two acquisition modes, we calculated the signal detectability using the channelized Hotelling observer (CHO) with Laguerre-Gauss (LG) channels. Our results show that spatial resolution of in-plane image in continuous mode was degraded due to the optical blurring of X-ray source. This blurring effect was reflected in aNPS, resulting in large *β* values. From a signal detectability perspective, the signal detectability in step-and-shoot mode is higher than that in continuous mode for small spherical signals but not large spherical signals. Although the step-and-shoot mode has disadvantage in terms of scan time compared to the continuous mode, scanning in step-and-shoot mode is better for detecting small signals, indicating that there is a tradeoff between scan time and image quality.

## Introduction

Mammography and digital breast tomosynthesis (DBT) systems are the most commonly used X-ray imaging modalities for breast cancer screening [1]. However, superimposed breast tissue is a major factor that degrades lesion detection performance in mammography due to the nature of the two-dimensional (2D) projection images [2]. Unlike mammography systems, DBT systems generate multiple projection data from a limited data acquisition range, and thus, the effect of overlapping breast tissues can be reduced, leading to improved detection performance [2, 3]. In DBT systems, it is important to find the optimal imaging protocol because lesion detection performance can be affected by several imaging parameters (e.g., system geometries, data acquisition modes, reconstruction algorithms, and X-ray source operations) [4, 5].

To improve the detection performance of a DBT system, many researchers have evaluated the effect of imaging parameters on DBT image quality [6–9]. Although the two acquisition modes are currently used for multiple projection data of DBT system, they acquired projection data using only one acquisition mode because commercial DBT systems use either step-and-shoot mode or continuous mode rather than both together [4]. The difference between two acquisition modes is optical blurring of the X-ray source (i.e., X-ray source motion) during data scanning [1, 4]. In the step-and-shoot mode, the X-ray source rotates along the arc path and stops stepwise at each angular spot to acquire projection data. On the other hand, the X-ray source moves during exposure in continuous mode. Since the X-ray source motion causes blurring of the lesion and breast anatomical background, not quantum noise, it is necessary to evaluate the effects of the two acquisition modes on the detection performance simultaneously under the same conditions.

For comparison of the two acquisition modes in DBT systems, modulation transfer function (MTF) and contrast were calculated using simulated phantoms [10, 11]. While they showed the blurring effect of X-ray source motion, there is a limitation as simulation studies that do not reflect physical factors (e.g., quantum noise, scattered radiation, and detector correlation). To overcome this limitation, some researchers used table-top experimental systems and compared DBT image quality using quantitative metrics such as MTF and contrast-to-noise ratio (CNR) [12, 13]. The results of these works experimentally showed noticeable differences between step-and-shoot mode and continuous mode. However, in the presence of anatomical background, the lesion detection performance of the DBT system was not reflected in the MTF and CNR measurements. To investigate the detection performance between two acquisition modes, in our previous work [14], we calculated the signal detectability of two acquisition modes using simulated breast phantoms. Although we showed optimal detection performance for the two acquisition modes according to the signal size, our simulation study still did not consider quantum noise, scatter radiation, and detector correlation.

The main contribution of this work is to investigate the effects of X-ray source motion on the signal detection performance of DBT systems. To accomplish this, we set a well-designed simulator and table-top experimental systems and generated the projection data of two acquisition modes using simulated breast phantom and BR3D breast phantom. After reconstruction, we measured in-plane MTF [15] and anatomical noise power spectrum (aNPS) [8] to evaluate the blurring effect of X-ray source motion. The optimal detection performance was calculated using a channelized Hotelling observer (CHO) with Laguerre-Gauss (LG) channels [16–18] to show how the blurring effect of the X-ray source motion has influence on signal detectability. The rest of this paper is organized as follows. In the Materials and Methods, we describe the simulation and experimental setup, sphere phantom, and breast phantom. Then, we explain how we calculated the quantitative metrics such as in-plane MTF, aNPS, and signal detection performance. The Results section gives the image quality assessment results and examples of reconstructed breast images. Finally, we present our findings, discussion, and conclusions in the Discussion and Conclusions section.

## Materials and methods

### System setup: Breast tomosynthesis simulator and table-top experimental system

**Fig 1:**
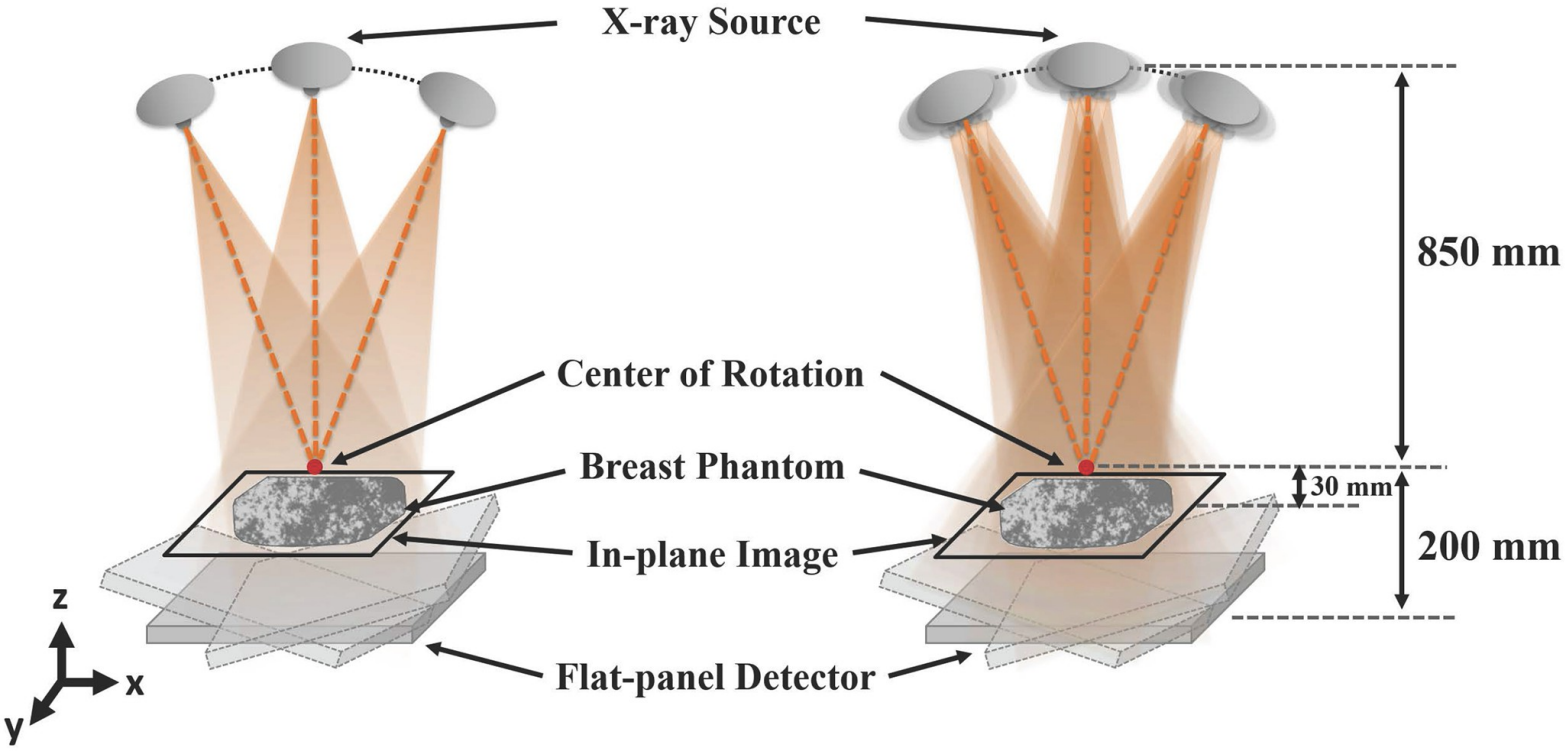
The geometry of digital breast tomosynthesis systems. (a) step-and-shoot mode and (b) continuous mode. The X-ray source and a flat-panel detector rotate simultaneously along the path within a limited angular range.

To acquire multiple projection data, we used a breast tomosynthesis system simulator with geometry parameters as described in Fig 1. The X-ray source and flat-panel detector are positioned at (0 mm, 0 mm, 850 mm) and (0 mm, 0 mm, -200 mm), respectively. The focal spot size was 0.3 × 0.3 mm^2^, and the detector cell size was 0.140 × 0.140 mm^2^. We used 17 × 17 source and detector lets to model the finite X-ray focal spot and detector cell [19]. The X-ray source and flat-panel detector rotate simultaneously, and projection data in step-and-shoot mode were generated from 25 views within a limited angular range of ±25° (Fig 1(a)). For the projection data in continuous mode, the angular span of each view was divided into 11 sub-angular samples (i.e., 11 view-lets) [20]. Afterward, the projection data of all view-let samples were averaged as shown in Fig 1(b). The projection data were filtered using a Hanning weighted ramp filter along the *x*-direction. For reconstruction, we performed four-fold Fourier interpolation and voxel-driven back-projection using linear interpolation [19]. The voxel size was 0.085 × 0.085 × 1.0 mm^3^, and the volume size (matrix size) of the reconstructed image was 8.5 × 8.5 × 64.0 mm^3^ (100 × 100 × 64 array) [17]. Table 1 summarizes the simulation parameters.

**Table 1:** Simulation parameters.

Parameter	Value
Source to iso-center distance	850 mm
Detector to iso-center distance	200 mm
Data acquisition angle	From −25° through 25° (50°)
Number of views	25
Detector cell size (array size)	0.140 × 0.140 mm^2^ (500 × 500)
X-ray focal spot size	0.3 × 0.3 mm^2^
Number of source and detector lets	17 × 17
Numver of view lets	11 (for continuous mode)
Reconstructed voxel size	0.085 × 0.085 × 1.0 mm^3^
Reconstructed volume size	8.5 × 8.5 × 64.0 mm^3^
Reconstructed matrix size	100 × 100 × 64
Diameter of sphere phantom	1 mm, 2 mm, 5 mm
Center of sphere and breast phantoms	(0.1 mm, 0.1 mm, -30 mm)
X-ray energy	30 keV monochromatic energy
Number of incident X-ray photons/detector cell	1.51 × 10^5^
Reconstruction algorithm	FDK

**Fig 2:**
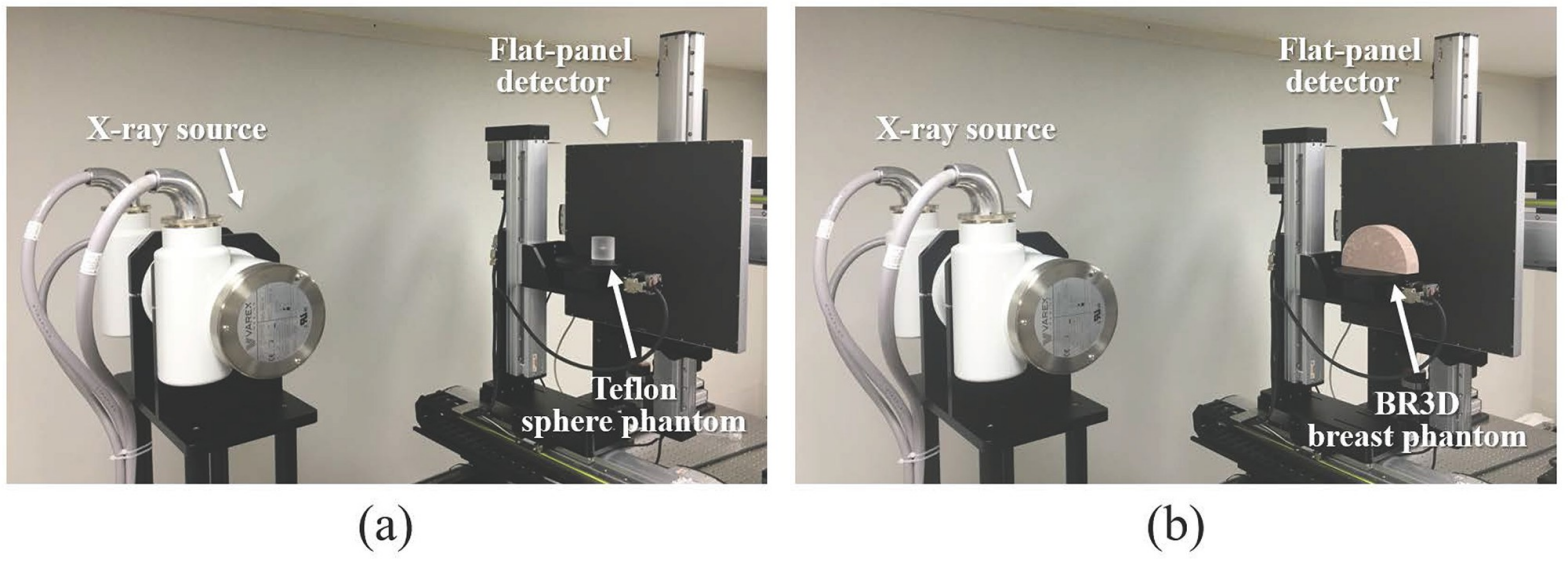
The table-top experimental system. (a) a Teflon sphere phantom and (b) a BR3D breast phantom.

For the experimental study, we used a table-top experimental system as shown in Fig 2. The table-top experimental system includes a generator (Indico IQ 50kW, CPI Communication & Medical Products Division, Georgetown, ON, Canada), a tungsten target X-ray source (VAREX RAD-14, VAREX X-ray Product, Salt Lake City, UT, USA) with a 0.3 × 0.3 mm^2^ focal spot, a 430 × 430 mm^2^ flat-panel detector (VIVIX-D 1717G, VIEWORKS, South Korea), and an object table. In the step-and-shoot mode, the X-ray source and flat-panel detector manually stopped at each angular spot, and the X-ray were irradiated. For the continuous mode, the X-ray source rotated approximately 20 mm during each projection data exposure. In both acquisition modes, we operated the X-ray source at 40 kVp, and the mean energy of the nominal 40 kVp incident spectrum was equivalent to about 30 keV monochromatic energy [21]. The detector was operated without binning mode, and the detector pixel size was 0.140 × 0.140 mm^2^ (3072 × 3072). The geometry of the table-top experimental system was used for simulation studies as summarized in Table 1. Table 2 summarizes the experimental parameters.

**Table 2:** Experimental parameters.

Parameter	Value
Source to iso-center distance	850 mm
Detector to iso-center distance	200 mm
X-ray focal spot size	0.3 × 0.3 mm^2^
X-ray energy	40 kVp (30 keV monochromatic energy)
Tube current-time product	400 mAs
Detector cell size	0.140 × 0.140 mm^2^
Detector array size	3072 × 3072 pixels
Data acquisition angle	From −25° through 25° (50°)
Number of views	25
Reconstruction algorithm	FDK

### Sphere phantoms

To measure the in-plane (*x*-*y* plane) MTF, we scanned a simulated and Teflon sphere phantoms (Engineering Laboratories, Oakland, NJ, USA) [22] using the simulator and the table-top experimental system, respectively. The simulated sphere object with 6.35 mm diameter was centered at (0.1 mm, 0.1 mm, -30 mm). We generated projection data and reconstructed the simulated sphere phantom using FDK algorithm with the simulation parameters shown in Table 1. For noise simulation, we used 1.51 × 10^5^ incident photons per detector cell and generated uniform noise following the Poisson distribution.

As shown in Fig 2(a), the Teflon sphere phantom was used for the experimental study. The Teflon sphere phantom included a 6.35 mm diameter Teflon ball embedded in a 50 mm diameter liquid silicone cylinder. The Teflon sphere phantom was placed 30 mm from the center of rotation (COR). To remove background trends from the reconstructed sphere phantom image, the background trends were modeled by a three-dimensional (3D) second-order polynomial function, and the coefficients of the polynomial function were estimated using the least-square fitting [23]. By subtracting the estimated background trends from the reconstructed sphere phantom image, we obtained sphere-only images that were used to measure the in-plane MTF.

We generated the ideal sphere phantom using the simulation. The volume (voxel) size was set as 8.5 × 8.5 × 64.0 mm^3^ (0.085 × 0.085 × 1.0 mm^3^). The image was first constructed a 1,000 × 1,000 × 640 array to avoid discretization error, and we performed down-sampling into a 100 × 100 × 64 array [24]. The ideal sphere phantom, the reconstructed sphere phantom image, and the detrended sphere phantom image were used to measure the in-plane MTF for the digital tomosynthesis system.

### Breast phantoms

#### Simulated breast phantoms

To model the breast anatomical background, we generated the breast anatomy structure using the power law spectrum [8, 25]:

$$\begin{array}{c} P\left(f\right)=\alpha /f^{\beta }, \end{array}$$ (1)

where *f* is the 3D radial frequency, *α* is a scaling factor, and *β* is the power law exponent. The parameter *α* is a binary attenuation coefficient because the breast anatomy structure mostly contains fibroglandular and adipose tissues [26, 27]. The exponent *β* is approximately 3, which has been reported in real clinical mammograms [28]. For the simulated breast volumes, a volume with 1,024 × 1,024 × 1,024 voxels of white Gaussian noise was first generated and filtered using the square root of 1/*f*^3^ [27, 29]. In the filter, the infinite value at zero frequency was replaced by twice the first non-zero frequency component [29]. Then, we extracted a central spherical volume with a diameter of 380 voxels from the filtered noise volume to avoid the boundary effect [30]. Since we considered a breast phantom with 50% volume glandular fraction (VGF), we sorted the voxel values of the spherical volume and replaced the top 50% voxel value with the attenuation coefficient of the fibroglandular tissue (0.0372 mm^−1^). The remaining 50% voxel values were set to the attenuation coefficient of the adipose tissue (0.0264 mm^−1^). Note that we used the attenuation coefficients at 30 keV monochromatic energy that correspond to the mean energy of the nominal 40 kVp incident spectrum [21, 31].

We used three spherical objects with diameters of 1 mm, 2 mm, and 5 mm as lesions within the anatomical background. The values of the breast phantom in the signal regions were replaced with the attenuation coefficient of the signals [17]. The attenuation coefficient of each signal was 0.0392 mm^−1^ at 30 keV monochromatic energy [31]. The voxel size of the simulated breast phantom was 0.11 × 0.11 × 0.11 mm^3^, and the volume size was 41.8 × 41.8 × 41.8 mm^3^. Note that the voxel size of the simulated breast phantoms (i.e., 0.11 mm) should be smaller than the magnified detector cell size at the COR (i.e., 0.1133 mm) to avoid discretization artifacts [32]. The simulated breast phantoms were located at (0.1 mm, 0.1 mm, -30 mm). To model quantum noise, we used 1.51 × 10^5^ incident photons per detector cell and generated uniform noise following the Poisson distribution, which is an equivalent dose used in single-view mammography (i.e., 3.4 mGy for breast with 50% VGF and 4 cm compressed breast thickness) [21].

The volume size of the reconstructed breast image was 32.3 × 32.3 × 64.0 mm^3^ (380 × 380 × 64 array) with a voxel size of 0.085 × 0.085 × 1.0 mm^3^. We extracted the central in-plane images (100 × 100 array) from the reconstructed breast image and used these to evaluate aNPS and signal detectability.

#### BR3D breast phantoms

We used the model 020 BR3D breast phantom (CIRS, VA, USA) for the experimental study [33]. We have eight BR3D breast background slabs. Each slab consists of 50% fibroglandular and 50% adipose tissues (i.e., 50% VGF) and has a unique swirling pattern. Among the eight slabs, four slabs were randomly selected to construct a BR3D breast phantom as shown in Fig 2(b). In this way, fifteen sets of BR3D breast phantoms with unequal slab order were constructed to obtain multiple backgrounds, and the size of each BR3D breast phantom was 100 mm × 180 mm × 40 mm (i.e., 4 cm compressed breast thickness).

For projection data, fifteen sets of BR3D breast phantoms were placed 30 mm from the COR and were scanned using the table-top experimental system with step-and-shoot mode and continuous mode. The X-ray source was operated at 40 kVp. To set the equivalent dose used in single-view mammography, DBT scan with approximately 400 mAs was performed in both acquisition modes (i.e., 3.4 mGy for breast with 50% VGF and 4 cm compressed breast thickness) [21]. In each reconstructed BR3D breast image, 200 regions of interest (ROI) images were randomly cropped. Each ROI image was a 100 × 100 array, and the pixel size was 0.085 × 0.085 mm^2^. The ROI images may overlap up to 10 pixels; the anatomical background of the signal region did not overlap. Furthermore, the ROI images without anatomical background were excluded from the data set. In each scan mode, we obtained a total of 3,000 ROI images. For signal-absent images, 1,500 ROI images were used, and another 1,500 ROI images were used for signal-present images.

For signal-present images, we inserted 1 mm, 2 mm, and 5 mm simulated spherical signals into signal-absent images. Since directly added signal and projection-based added signal would denature the intrinsic properties of the anatomical background where the signal is located, we used the modulation on tissue and the sphere signal boundary approach [34, 35]. We determined modulation functions for the anatomical background and spherical signal and inserted the modulated signal into the modulated anatomical background. In this work, signal-present and signal-absent image pairs were used to calculate the signal detectability.

### Image quality assessment

#### In-plane modulation transfer function

To show the effect of X-ray source motion on spatial resolution, we calculated in-plane MTF using reconstructed images of simulated sphere and Teflon sphere phantoms. In our previous work [15], we proposed an inverse filtering approach to measure in-plane MTF for a DBT system and validated the effectiveness of the proposed method using a simulated sphere and Teflon sphere phantoms. Since we used the inverse filtering approach in this work, we briefly review the approach for readers.

Table 3 summarizes the inverse filtering approach to measure in-plane MTF for a DBT system. In Step 1, the reconstructed sphere phantom can be expressed by a triple convolution operator. Then, in accordance with the central slice theorem, in-plane PSF and in-plane MTF are calculated in Step 2. The inverse filter 1/*S*(*f*_*x*_, *f*_*y*_, *f*_*z*_) amplifies the true values of *M*(*f*_*x*_, *f*_*y*_) when *S*(*f*_*x*_, *f*_*y*_, *f*_*z*_) has small values at certain frequencies. To resolve this problem, we applied pseudo inverse filtering to estimate the in-plane MTF as shown in Step 3. Although the estimation errors are reduced in Step 3, missing data are produced in the *S*_inv_(*f*_*x*_, *f*_*y*_, *f*_*z*_) term due to the zero values as shown in line 6. In Step 4, we used the Laplacian operator to correct the residual errors. In this work, the scaling factor *γ* was experimentally set to 0.1, and the iteration was terminated when the gradient of the total variation was lower than the error tolerance 4 × 10^−3^. Finally, we can measure the in-plane MTF, *M*^(*i*+ 1)^(*f*_*x*_, *f*_*y*_ = 0).

**Table 3:** Inverse filtering approach to measure in-plane MTF.

1: *Step 1*. *Reconstructed sphere phantom expressed by triple convolution*
2: *k*(*x*, *y*, *z*) = *s*(*x*, *y*, *z*) * * * *p*(*x*, *y*, *z*) ↔ *K*(*f*_*x*_, *f*_*y*_, *f*_*z*_) = *S*(*f*_*x*_, *f*_*y*_, *f*_*z*_) × *P*(*f*_*x*_, *f*_*y*_, *f*_*z*_) where *k*:reconstructed sphere phantom, *s*: ideal sphere phantom, *p*: 3D point spread function, *: convolution operator, ↔: Fourier transform
3: *Step 2*. *Applied central slice theorem*
4: $p\left(x,y,z=0\right)\Leftrightarrow \int P(f_{x},f_{y},f_{z})\;df_{z}=\int \frac{K(f_{x},f_{y},f_{z})}{S(f_{x},f_{y},f_{z})}\;df_{z}=M\left(f_{x},f_{y}\right)$ where *p*(*x*, *y*, *z* = 0): in-plane PSF, *M*(*f*_*x*_, *f*_*y*_): in-plane MTF
5: *Step 3*. *Pseudo inverse filtering*
6: $M(f_{x},f_{y}=0)\approx \int K(f_{x},f_{y},f_{z})\;S_{\text{inv}}(f_{x},f_{y},f_{z})\;df_{z}{|}_{f_{y}=0}$ where $S_{\text{inv}}\left(f_{x^{\prime }},f_{y^{\prime }},f_{z^{\prime }}\right)=\{\begin{array}{cc} & 1/S\left(f_{x^{\prime }},f_{y^{\prime }},f_{z^{\prime }}\right),\;\;\;\text{if}\;\;\left|S\left(f_{x^{\prime }},f_{y^{\prime }},f_{z^{\prime }}\right)\right|>\text{threshold}\\ & 0,\;\;\;\;\;\;\;\;\;\;\;\;\;\;\;\;\;\;\;\;\;\;\;\;\;\;\;\;\text{otherwise}. \end{array}$
7: *Step 4*. *Residual error reduction uaing the Laplacian operator*
8: *M*^(*i*+1)^(*f*_*x*_, *f*_*y*_ = 0) = *M*^(*i*)^(*f*_*x*_, *f*_*y*_ = 0) − *γ*Δ*M*^(*i*)^(*f*_*x*_, *f*_*y*_ = 0) where *i*:iteration number, *γ*: scaling factor, Δ: Laplacian operator
9: Estimated in-plane MTF = *M*^(*i*+1)^(*f*_*x*_, *f*_*y*_ = 0)

#### Anatomical noise power spectrum and exponent *β* value

To show the anatomical noise power distribution over spatial frequencies, we calculated aNPS for each scan mode using the reconstructed breast images of simulated and BR3D breast phantoms. First, the ensemble mean value was computed using 500 signal-absent reconstructed in-plane images. To yield zero-mean signal-absent images, we subtracted the ensemble mean value from each image. To suppress the appearance of artifacts from spectral leakage, we applied the Hanning tapering window in Eq (2) to each zero-mean signal-absent image [8, 26].

$$\begin{array}{c} W\left(r\right)=\{\begin{array}{cc} & 0.5+0.5\text{cos}(\pi r/D),\;\;\;\text{for}\;\;r\leq D\\ & 0\;\;\;\;\;\;\;\;\;\;\;\;\;\;\;\;\;\;\;\;\;\;\;\;\;\;\;\;\;\;\;\;\;\;\;\text{for}\;\;r>D, \end{array} \end{array}$$ (2)

where *r* is the radial distance from the center, and *D* is half of the image width. The 2D aNPS was calculated by ensemble averaging the square of the magnitude of the discrete Fourier transform of each tapered image. We performed radial averaging of the 2D aNPS, yielding 1D aNPS, and applied the natural logarithm to the radially averaged 1D aNPS to accentuate different noise structures. To compute the exponent *β* value in Eq (1), we performed linear regression on the logarithm-applied 1D aNPS over frequency ranges and selected the ranges that maximize the fit of the linear regression model determined by the coefficient of determination (i.e., *R*^2^) [8].

#### The mathematical model observer

To evaluate the signal detectability of both scan modes, we conducted binary detection tasks with signal-known-exactly (SKE) and background-known-statistically (BKS) schemes. Two hypotheses (i.e., *H*_0_ for signal-absent and *H*_1_ for signal-present) are considered as follows:

$$H_{0}:\;g=b_{\text{sa}}+n,$$ (3)

$$H_{1}:\;g=b_{\text{sp}}+n,$$ (4)

where vector **b**_**sa**_ is the anatomical background, and vector **b**_**sp**_ is the anatomical background including the signal. Vector **n** is the reconstructed noise, and vector **g** is the reconstructed in-plane image.

**Fig 3:**
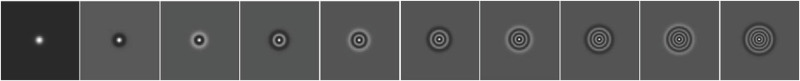
Exampled 10 number of LG spatial channel images with *a*_*u*_ = 11 pixels (left: *p* = 0, right: *p* = 9).

For mathematical model observer study, we used CHO with LG channels (LG CHO), and the LG CHO approximates the performance of the Hotelling observer. [16–18, 36]. We generated LG channels using the following Gaussian functions:

$$\begin{array}{c} u_{p}\left(r|a_{u}\right)=\frac{\sqrt{2}}{a_{u}}\text{exp}(\frac{-\pi r^{2}}{a_{u}^{2}})L_{p}(\frac{2\pi r^{2}}{a_{u}^{2}}), \end{array}$$ (5)

with the Laguerre polynomial function

$$\begin{array}{c} L_{p}\left(x\right)=\sum_{k=0}^{p}{\left(-1\right)}^{k}(\frac{p}{k})\frac{x^{k}}{k!}, \end{array}$$ (6)

where **r** is a 2D spatial coordinate, *a*_*u*_ is the width of the Gaussian function, and *p* is the polynomial order. To approximate the performance of the Hotelling observer, the Gaussian width *a*_*u*_ should be set to maximize the signal detection performance of LG CHO. Since the degree of signal blurring differs depending on which scan mode is used, even if the signal size is known, it is necessary to find the *a*_*u*_ that maximizes the signal detectability. We performed brute-force searching within the range of 3—60 pixels, and the optimal value of *a*_*u*_ was proportional to the diameter of the signal. For the LG channel number, the signal detectability of LG CHO was saturated when the LG channel number was 10 or more, and thus we used 10 LG channels to assess the detection performance of both scan modes. Fig 3 shows an example 10 LG channel images.

The CHO uses channelized image vector instead the image vector, and thus the CHO is beneficial for reducing the data dimensionality of **g**. We applied the channel matrix **T** to **g**, which resulted in a channelized image **v** expressed as follows:

$$\begin{array}{c} v=Tg. \end{array}$$ (7)

For the LG CHO, we implemented Matrix **T** of the LG channels using discrete sample values from Eq (5). The template of CHO, **w**_**v**_, and decision variable *t*_*j*_ can be computed by

$$\begin{array}{cc} & w_{v}={K_{v}}^{\text{-1}}\Delta v_{s}, \end{array}$$ (8)

$$\begin{array}{cc} & t_{j}={w_{v}}^{t}v_{j},\;\;\;\;\;\;\;\;\;\;\;\;\;\;\;\;\;\;j=\text{0,}\;\text{1,} \end{array}$$ (9)

where **K**_**v**_ is a covariance matrix, and **v**_1_ and **v**_0_ are channelized signal-present and signal-absent images, respectively. The Δ**v**_**s**_ is the mean difference between the two channelized images. To compute the covariance matrix **K**_**v**_, we averaged the covariance matrices of **v**_1_ and **v**_0_.

To determine the signal detection performance, we calculated the SNR_t_ value as a figure of merit given by [37]:

$$\begin{array}{c} {\text{SNR}}_{\text{t}}=\frac{<t_{\text{1}}>-<t_{\text{0}}>}{\sqrt{\frac{1}{2}\left(\sigma_{t_{\text{1}}}^{2}+\sigma_{t_{\text{0}}}^{2}\right)}}, \end{array}$$ (10)

where <∘> is an expectation operator. The parameters $\sigma_{t_{1}}$ and $\sigma_{t_{0}}$ denote the standard deviations of signal-present (*t*_1_) and signal-absent (*t*_0_) decision variables, respectively.

To train the LG CHO, we used 200 image pairs and estimated the covariance matrix **K**_**v**_ Another 200 independent image pairs were used to calculate Δ**v**_**s**_ For observer testing, we computed decision variables *t*_*j*_ using another 100 independent image pairs. For observer training variability, we used three independently generated training data sets and trained three model observers. Then, the three SNR_t_ from three model observers were averaged. For case variability, the error bars of SNR_t_ were estimated by bootstrapping the decision variable 1,000 times [38].

## Results

**Fig 4:**
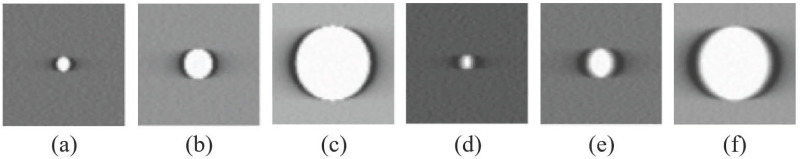
Reconstructed images of spherical signals in (a—c) step-and-shoot mode, and (d—f) continuous mode. Signal diameters are (a, d) 1 mm, (b, e) 2 mm, and (c, f) 5 mm. The display window is set by [min. max.] mm^−1^.

**Fig 5:**
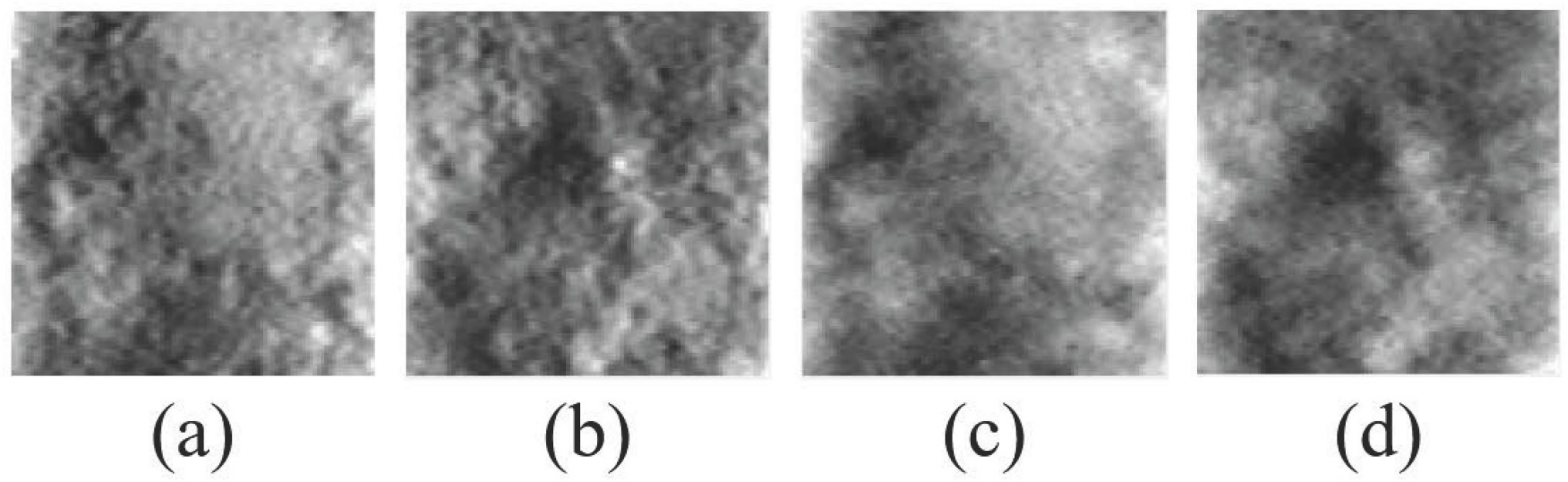
Examples of reconstructed simulated breast phantom without spherical signal. (a) and (b) are reconstructed images using projection data in step-and-shoot mode, and (c) and (d) are corresponding images using projection data in continuous mode. The display window is set by [min. max.] mm^−1^ to visualize the background structures more clearly.

**Fig 6:**
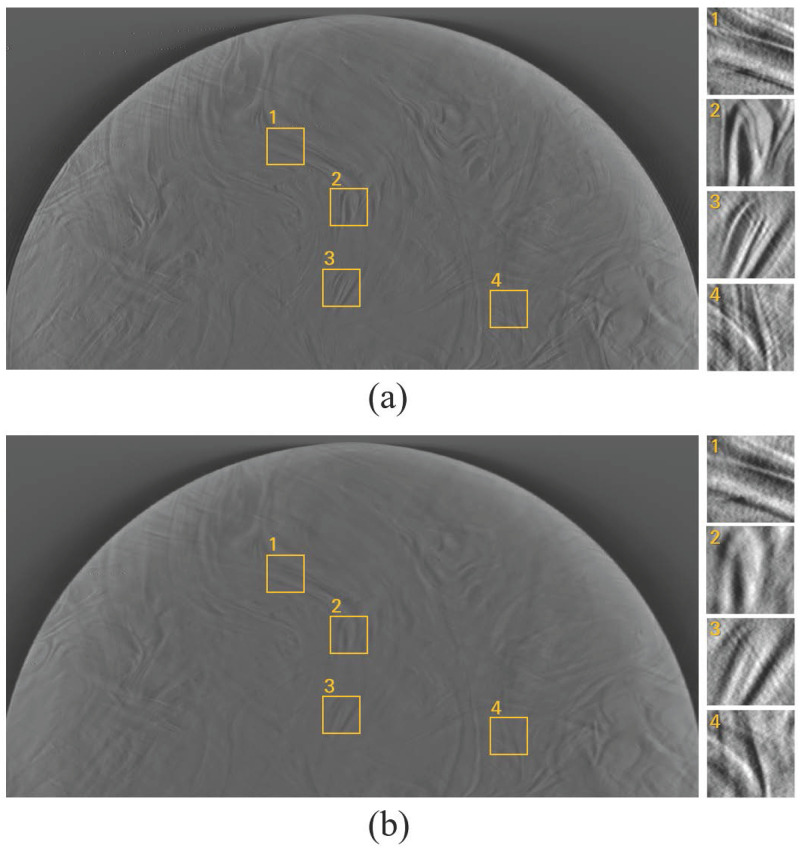
Examples of reconstructed BR3D breast phantom without spherical signal. (a) is a reconstructed image using projection data in step-and-shoot mode, and (b) is the corresponding image using projection data in continuous mode. Four 100 × 100 images are cropped to show the detailed anatomical background (indicated by the yellow box). The display window is set by [min. max.] mm^−1^ to visualize the background structures more clearly.

Fig 4 shows the reconstructed images of simulated spherical signals in step-and-shoot mode and continuous mode. Compared to the step-and-shoot mode, signal blurring is clearly visualized in continuous mode due to the X-ray source motion as shown in Fig 4(d)–4(f). Fig 5 shows examples of the reconstructed images of simulated breast phantoms. As in the signal images shown in Fig 4, the anatomical background in continuous mode (Fig 5(c) and 5(d)) is more blurry than that in step-and-shoot mode (Fig 5(a) and 5(b)). As shown in Fig 6, the anatomical background of the BR3D breast phantom is also affected by the X-ray source motion. To show the detailed anatomical background, we cropped four 100 × 100 images (indicated by the yellow box). The blurring effects in continuous mode can be clearly observed in the cropped images.

**Fig 7:**
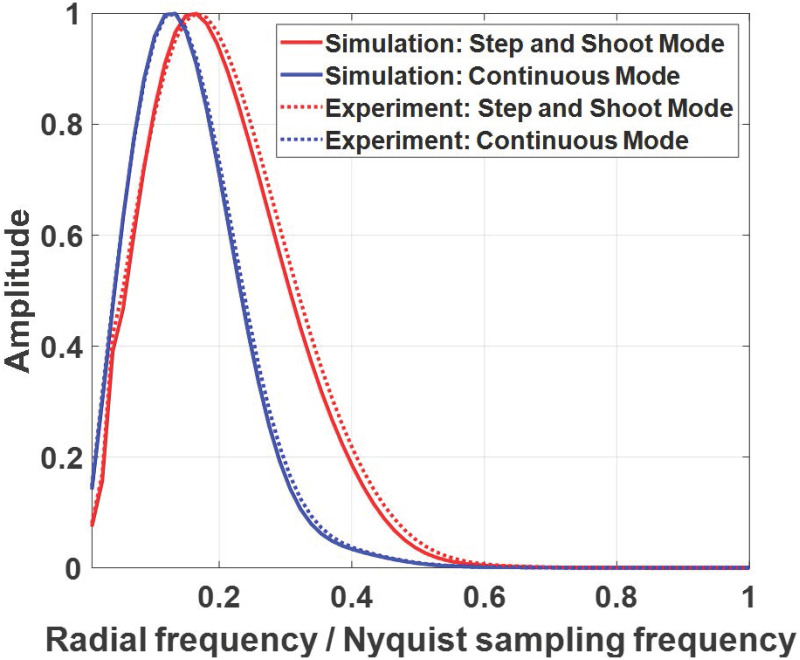
In-plane MTFs in step-and-shoot mode (indicated by the red color) and continuous mode (indicated by the blue color) using the simulated sphere phantom (indicated by the solid line) and experimental Teflon sphere phantom (indicated by the dashed line).

Fig 7 shows measured in-plane MTFs in step-and-shoot mode (indicated by the red color) and continuous mode (indicated by the blue color). Compared to step-and-shoot mode, the spatial resolution of the in-plane image in continuous mode was degraded due to the X-ray source motion. This blurring effect is reflected in reconstructed signal and anatomical background images as shown in Figs 4–6. Furthermore, the measured in-plane MTFs using the Teflon sphere phantom (indicated by the dashed line) show agreement with those using the simulated sphere phantom (indicated by the solid line), which demonstrates that the simulator well models the optical blurring of the X-ray source.

**Fig 8:**
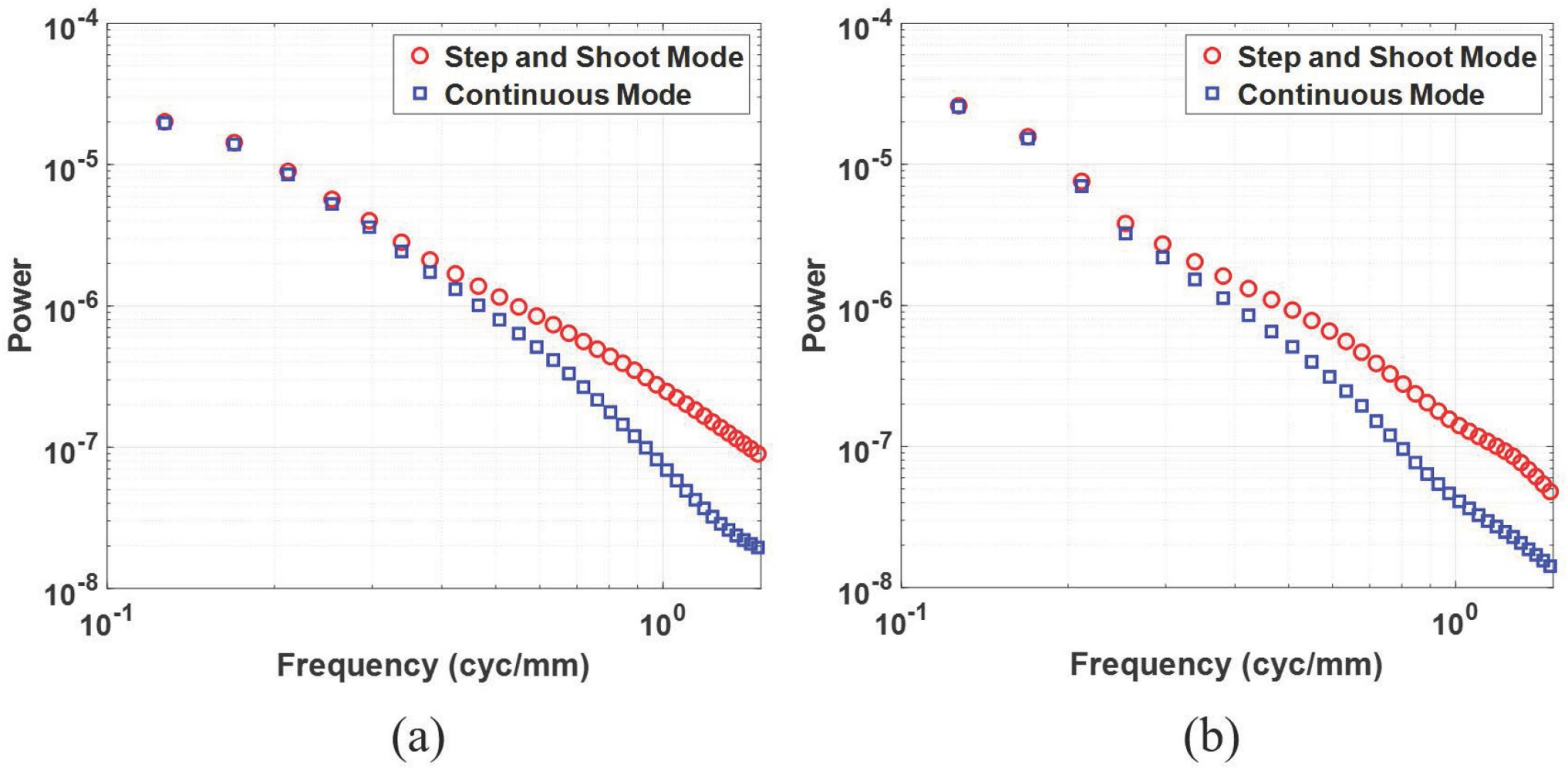
Anatomical noise power spectrum from the reconstructed in-plane images using (a) simulated breast phantom and (b) BR3D breast phantom.

Fig 8 illustrates radially averaged aNPSs using reconstructed in-plane images with simulated breast phantom and BR3D breast phantom. Although not presented in this paper, the signal power was concentrated under 1.5 cyc/mm, and thus the radially averaged aNPSs are plotted up to the same value. Compared to the aNPS for step-and-shoot mode (indicated by the red circle marker), the aNPS for continuous mode (indicated by the blue square marker) is blurred by the X-ray source motion, and the aNPS gap between both acquisition modes increases as the frequency increases. Using the radially averaged aNPS, we estimated the exponent *β* values, as reported in Table 4. We fitted the aNPS in the range of 0.3—0.7 cyc/mm. This is because the *R*^2^ value was larger than 0.99 [26]. The high frequency components of aNPS for continuous mode are suppressed by the X-ray source motion, which increases the slope of the logarithm-applied radially averaged aNPS and results in a higher *β* values. Note that, smaller *β* values were regarded as an indicator of better detectability. The trends of aNPS blurring and *β* values in both simulation and experimental studies are similar, indicating that the simulator well reflects the real X-ray source motion, as with the in-plane MTF.

**Table 4:** *β* values of the anatomical NPS.

Scan mode	Simulated breast phantom	BR3D breast phantom
Step and shoot mode	2.16	2.24
Continuous mode	2.96	3.10

**Fig 9:**
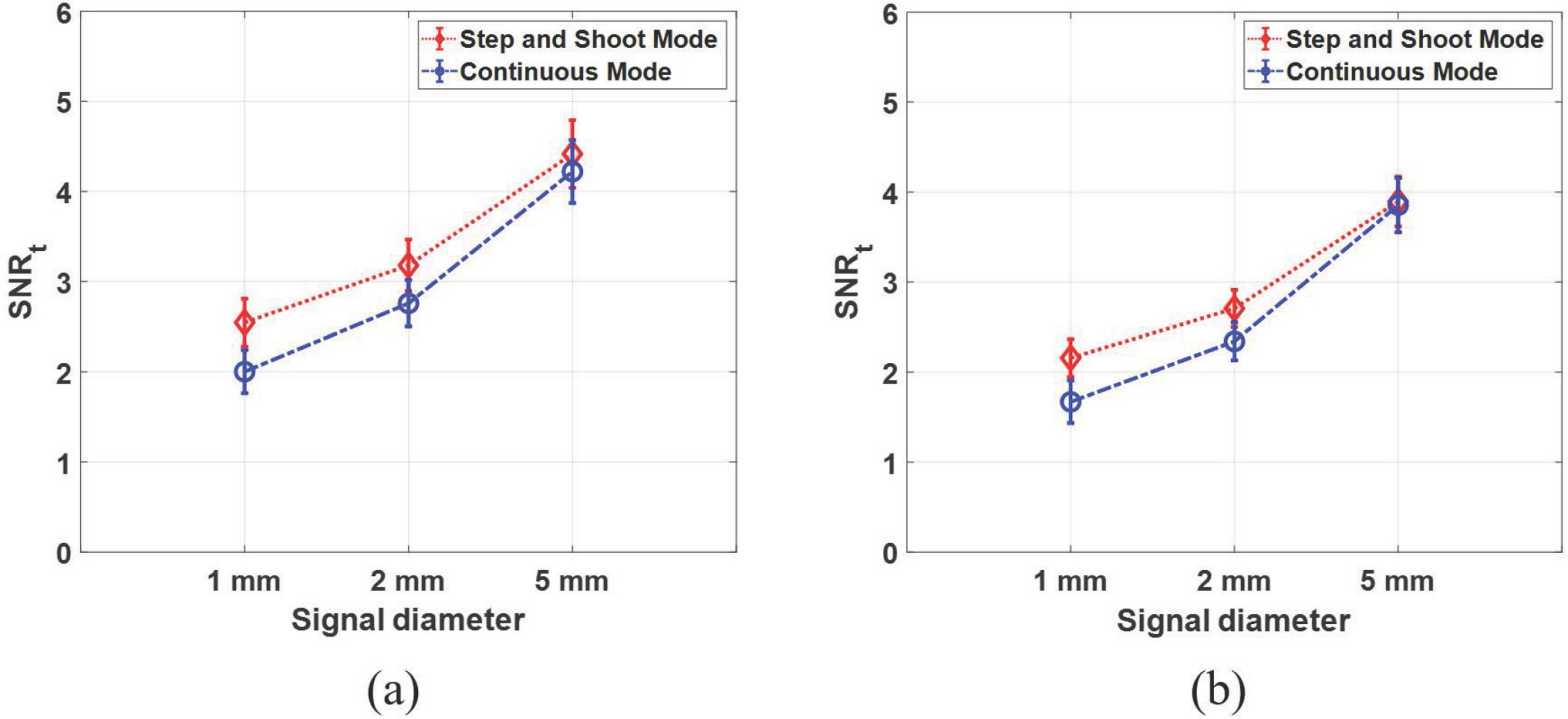
SNR_t_ values of model observers for reconstructed images of (a) simulated breast phantom and (b) BR3D breast phantom. The SNR_t_ values of model observers with 95% confidence intervals.

Fig 9 shows the SNR_t_ values of LG CHO with 95% confidence intervals in step-and-shoot mode (indicated by the red dotted line and diamond marker) and continuous mode (indicated by the blue dashed dot line and circle marker). In Fig 9(a), the SNR_t_ in step-and-shoot mode is higher than that in continuous mode for small spherical signals (i.e., 1 mm, and 2 mm), indicating that using the step-and-shoot mode is more beneficial when detecting small spherical signals in DBT systems. The X-ray source motion makes the signal and anatomical noise power more blurred as observed in Figs 7 and 8, introducing lower signal detection performance. However, when the signal diameter increases (i.e., 5 mm), the signal detectability of both step-and-shoot mode and continuous mode are statistically similar because aNPSs of both acquisition modes are similar at low frequency region where a large signal has more energy. In other words, the X-ray source motion does not have a significant effect on the detection performance for large signals, which is not predicted by the results of in-plane MTF and *β* values. As shown in Fig 9(a) and 9(b), the overall SNR_t_ trends of experimental study are similar with that of simulation study, but the level decreases. The simulation study did not consider the effect of X-ray scatter. Since the scatter radiation degrades the contrast of reconstructed in-plane images, the level of detection performance with scatter radiation (experimental results) decreases compared to that without scatter radiation (simulation results).

**Fig 10:**
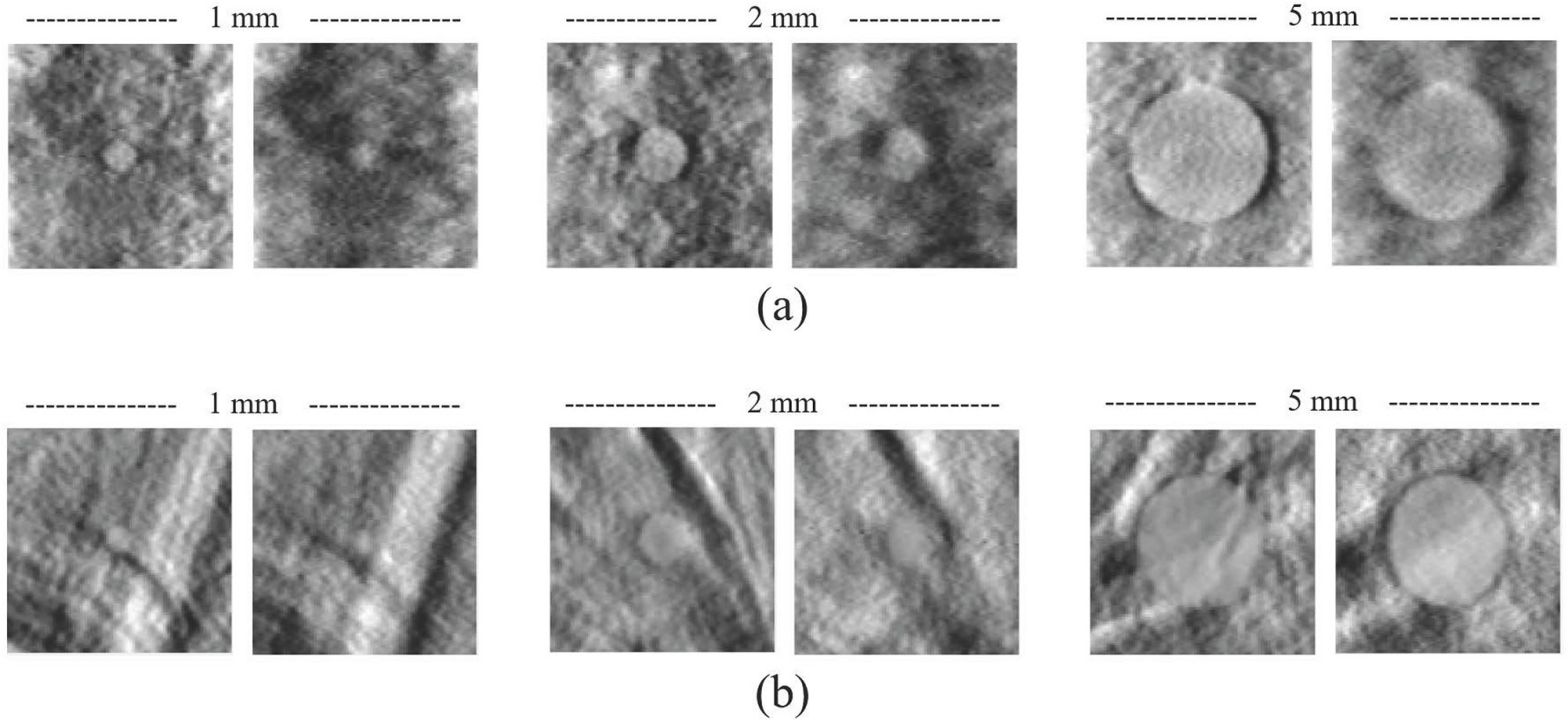
Examples of reconstructed images with spherical signals from (a) simulated breast phantom and (b) BR3D breast phantom. In each image set, the left image was obtained in step-and-shoot mode, and the right image was obtained in continuous mode. The display window is set by [min. max.] mm^−1^ to visualize the background structures more clearly.

For qualitative comparison, we sampled a few reconstructed in-plane images with spherical signals used in this work, as shown in Fig 10. It can be clearly observed that both the signal and anatomical background are blurred by the X-ray source motion. Furthermore, as predicted from Fig 9, using a step-and-shoot mode can help improve the signal detection performance for small spherical signals. In the case of a large spherical signal, the signal detectability appears qualitatively similar, although the blurring effects are observed in both signal and anatomical background.

## Discussion and conclusions

We investigated the effects of the optical blurring of X-ray source on DBT image quality using a well-designed simulator and table-top experimental systems. Using simulated and BR3D breast phantoms, we reconstructed breast images using projection data in step-and-shoot mode and continuous mode, and computed the in-plane MTF, aNPS, and signal detectability to compare the image quality of both scan modes. Our results showed that spherical signal and anatomical background blurring are visualized in continuous mode due to X-ray source motion, which is measured by in-plane MTF and aNPS. For signal detectability perspective, the step-and-shoot mode provides better signal detectability for small spherical signals.

We considered two dominant breast anatomy (i.e., fibroglandular and adipose tissues) and spherical signals, both concentrated in low frequency regions. At the low frequency regions, the anatomical noise is dominant over quantum noise [9, 32]. Thus, in a lower dose regime than that used in this work, even if the quantum noise is more severe, the trends of signal detection performance would be expected to be similar.

Although real breast tissues have preferred orientations [39], this is not reflected in the simulated breast phantom. Unlike the simulated breast phantom, the BR3D breast phantom has a preferred orientation for each region due to material swirling. Therefore, regions with similar orientations as the X-ray source motion direction (e.g., image 1 in Fig 6) have different blurring effects compared to regions that have a perpendicular direction (e.g., image 2 in Fig 6). Although we consolidated multiple ROIs of reconstructed BR3D breast images that have different orientations, we reflected breast tissue orientation through the BR3D breast phantom. Investigating the effect of specific orientations of breast tissue on signal detectability would be an interesting future research topic.

In this work, we investigated the effects of X-ray source motion using both simulation and experimental systems. Nevertheless, it does not fully include real physical factors. (1) We set the system geometry with 850 mm source to iso-center distance (SID) and 200 mm detector to iso-center distance (DID) to match the operating range of the table-top experimental system. Although not presented in this paper, we also investigated the effect of X-ray source motion using a simulator with a similar geometry to a real breast tomosynthesis system (e.g., 605 mm SID and 70 mm DID). Regardless of the system geometry, the overall trends of in-plane MTF, anatomical NPS, and SNR_t_ are similar to the results of this work. (2) The breast tissue orientation was reflected through the BR3D breast phantom, but the signals with more complex shapes and preferred orientations were not considered in this work [27]. Since LG CHO provides suboptimal signal detection performance in that it is not optimal for clinically relevant signals, it is necessary to evaluate signal detectability using a model observer with other channels (e.g., partial least squares (PLS) channel) [40]. This would be the subject of our future research topic. (3) Our results showed that using the step-and-shoot mode leads to improvement in small signal detection performance, but patient motion is not considered. Although the breast is compressed and fixed by the compression paddle device, the advantage of the step-and-shoot mode in small signal detectability would be reduced when the patient motion occurs.

From our results, scanning in step-and-shoot mode is better for detecting small signals. However, compared to the continuous mode, the step-and-shoot mode has the disadvantage that the scan time is longer and may be affected by the patient motion. There is a tradeoff between scan time and image quality, and thus, developing a deblurring algorithm and applying it to scan data of continuous mode would be a meaningful research topic.

In conclusion, we evaluated the DBT image quality using in-plane MTF, aNPS, and signal detectability to show the effect of optical blurring of X-ray source on reconstructed DBT image. We first investigated the DBT image quality using a well-designed DBT simulation. To validate the simulation results, we conducted an experimental study using a table-top experimental system and BR3D breast phantom. We observed that the X-ray source motion introduced blurred in-plane MTF and aNPS, yielding lower signal detectability for small spherical signals, not for large signals. This finding indicates that the choice of DBT scan mode is important for small spherical signal detection performance in the presence of anatomical background.

## Supporting information

S1 File SNR_*t*_ by each of three mathematical model observers. SNR_*t*_ values by each of the three mathematical model observers for reconstructed images of simulated and BR3D breast phantoms.
(XLSX)
